# Sex, relationships and ‘everyday psychology’ on British magazine problem pages, c. 1960–1990

**DOI:** 10.1136/medhum-2022-012497

**Published:** 2022-12-22

**Authors:** Tracey Loughran

**Affiliations:** History, University of Essex, Colchester, Essex, UK

**Keywords:** history, cultural history, medical humanities

## Abstract

The later decades of the 20th century saw dramatic changes in sexual attitudes and behaviour in Britain: rates of divorce and remarriage increased; premarital sex and illegitimacy became more common, even as the pill and legal abortion opened up new reproductive choices; and following on from the decriminalisation of homosex, liberation movements began to celebrate gay lives. These shifts generated new possibilities, but often entailed much inner turmoil. The same period witnessed an unprecedented flourishing of professional and popular psychological expertise. Influential social and cultural theorists have argued that the intertwined rise of “permissiveness” and therapeutic culture caused an important shift in the ethical dimensions of modern life, in which citizens and subjects came to idolise self-realisation over the public good.

This article uses women’s magazine problem pages, exploring the role of advice columnists on and off the page, to examine the intersections of “permissiveness” and the psychologisation of everyday life. Millions looked to agony aunts in mass-market women’s magazines to help them negotiate new emotional and sexual worlds. As purveyors of counsel, but not (usually) formally trained counsellors, magazine advisors worked with the new languages and concepts of psychological expertise and disseminated them to avid readers.

Across this period, problem pages demonstrated greater openness towards sex and displacement of morality from external standards to the individual. However, advisors also continued to emphasise self-control and responsibility, and to provide practical guidance that took at best a superficially psychological veneer. These trends were underpinned by a model of sex as an essential part of loving, stable relationships, and the (largely unexpressed) notion that such relationships were essential to social functioning. In the woman’s world of the magazine, before and beyond the 1980s, the problem page does not show the rise of individualism or the pursuit of pleasure above all else.

## Introduction

‘We’ve been married six years and for the past three my husband has preferred masturbation to sex with me’, opened a letter published on the *Woman’s Own* problem page (Anon 1979a). The correspondent went on to explain that as a result, she could ‘hardly bear him to touch me and have to force myself to have sex with him about once every two to three months’. She and her husband realised that ‘we need help if we’re to stay together’ but each blamed the other for the ‘lack of sex’: she did not believe that he had given up masturbation, while he accused her of ‘coldness’. There seemed no point visiting a marriage counsellor when they could not agree on the cause of the problem: ‘We are stuck at this point and I can’t see any way out’.

The response of Mary Grant, *Woman’s Own*’s resident agony aunt, situated this couple’s dilemma within the wider context of contemporary sexual mores, therapeutic culture and legal reforms:

Now so much sexual and marital help is available, we all need a new approach to the whole business of getting help if we’re to make the most of it. The pity is that most people’s approach to marriage problems, sexual or otherwise, is based on the old legal attitude to divorce; then there was one guilty partner to a marriage breakdown, and one innocent one, and everything was geared to proving who was to blame. Well, this idea of right and wrong was never applicable to the complexities of human relations and the present divorce laws recognise it, but we still cling to the old idea (Grant 1979a).

But, she went on, it was irrelevant ‘who began the trouble’: ‘both partners always contribute to the good bits and the sad bits of a marriage’, both partners ‘keep the problem in being, and both of you suffer from it’, so it was also up to both partners to ‘want to put things right and stay happily together’. The ‘next step towards a solution’ was ‘going to see a marriage counsellor’, and although someone had to make the first move to organise this, it would help both parties. Ultimately, the person with ‘most hope and love and good sense’ was the one who ‘seeks a way out of the problem—which you’ve done by writing to me’.

In only a few lines, this letter and response opens out the interconnectedness of changing approaches to sex and relationships on the one hand, and therapeutic culture on the other, in late 20th century Britain. The correspondent’s use of frank sexual language, the magazine’s willingness to print the letter and the shared belief of the woman, her husband and Mary Grant in the centrality of sexual satisfaction to happy marriages, and in the legitimacy of actively pursuing that end, with outside help if required—all situate this problem in the long “permissive moment” following the raft of liberalising legislation at the end of the 1960s, and the social and sexual turmoil that preceded it (Weeks 2017, 272–391; Mort 2011). The letter-writer and her husband had to negotiate through a maze of rapidly shifting social attitudes that included the heightened value placed on romantic love, softer stances towards illegitimacy, premarital and extramarital sex, adjustment to the effects of the contraceptive pill and the rise in women’s full-time employment outside the home, and the claims of liberation movements to autonomy and visibility. It is perhaps no wonder they had trouble finding their way and needed some help.

And, as Mary Grant noted, the menu of ‘help’ available was longer than ever before. The postwar decades witnessed an unprecedented flourishing of psychological expertise across the UK, in forms as diverse as the Marriage Guidance Council, community counselling initiatives, volunteer-run helplines and the services provided by national charities like MIND, not to mention general practitioners trained in the Balint method, social workers who received psychological education as part of their standard qualifications and psychotherapists and psychoanalysts in private practice (Lewis, Clarke, and Morgan 1991; Crossley 2005; Osborne 1993, 112–27; Thomson 2006, 251–88). The possibilities of so much choice might well be bewildering.

In the midst of this chaos, magazine advice columnists provided guidance to those who did not know where else to turn. The problem pages in these publications had enormous potential reach and influence. In the early 1960s, over 50 million British women read a women’s weekly and 34 million read a monthly. By 1987, these numbers had declined to nearly 24 million and nearly 40 million, respectively—still a substantial readership (Ballaster et al. 1991, 111). There is disagreement about whether this influence was in itself ‘a good thing’. Older traditions of scholarship on women’s magazines, scaffolded by 1970s socialist feminism, castigated advice columns for their failure to recognise that ‘women’s problems may have political origins, be politically structured or politically transformable’ (Ballaster et al. 1991, 146–7; Winship 1987, 77–80). Against this,Adrian Bingham (2012, 52), highlights the potential of advice columns to challenge conservative attitudes to gender, sexuality and pleasure, and argues that by the 1960s, newspaper problem pages had ‘contributed to significant shifts in British sexual culture’. As similar ‘cultural intermediaries’, magazine advisors likewise held considerable power to shape popular understandings of sex and relationships (McKay 2008).

Magazine advice columnists were also crucial arbiters in popular psychological culture. In the postwar decades, psychological norms and languages came to extend beyond ‘the consultation, the interview, the appointment room’ to become ‘part of the staple fare of the mass media of communication’ (Rose 1989, xii, 208, 214; Giddens 1991, 70–108; Giddens 1992, 30). As purveyors of counsel, if not formally trained counsellors, they worked with the new languages and concepts of psychological expertise and disseminated them to millions upon millions of avid readers. On magazine problem pages, people ‘met the language and assumptions of psychology on a regular basis’ without ‘actively seeking it’ (Thomson 2006, 4). Magazine problem pages therefore offer an ideal lens through which to examine the intersections of “permissiveness” and the psychologisation of everyday life.

For scholars such as Philip Rieff, Christopher Lasch, and Frank Furedi (Furedi 2004; Lasch 1979; Rieff 1966), therapeutic culture and ‘“permissiveness” are hopelessly intertwined in modern societies that celebrate self-obsessed individuals, freed from traditional authority and moral constraint, in contrast to the publicly oriented conceptions of the ideal self that characterised earlier societies (Wright 2008). On the surface, there are some similarities between this analysis and social theorist Nikolas Rose’s contention that in late modern societies, the psychologisation of everyday life sees the transposition ‘from an ethical to a psychological register’ of ‘the problems of defining and living a good life’ (Rose 1989, xiii). However, for Rose the therapeutic culture of the 1960s marked not the liberation of the self, but the creation of new forms of subjectivity through the invention of techniques of self-introspection, modes of self-presentation and vocabularies of the emotions. These new forms of self-government might promise ‘autonomy and success’ but at the price of constant scrutiny, evaluation and self-doubt (Rose 1989, 239, 115–16).

These analyses propose an important shift in the ethical dimensions of modern life, in which the “permissive moment” accelerated the rise of therapeutic culture and intensified the forces within it that led subjects to idolise self-realisation over the public good. More recent historical scholarship disputes these claims. Rusterholz (2019, 2021, 2022) has shown that mid-century sexual counselling called on individuals to work actively, stoically and responsibly towards achievement of the emotional openness perceived as necessary to mutual sexual satisfaction in stable heterosexual relationships. This research confirms and extends the analysis of Chettiar (2016), who sees the state-sponsored expansion of marriage counselling and debates on divorce reform as two sides of the same coin; both demonstrate the identification of romantic and sexual relationships, underpinned by psychologised concepts of emotional health, as essential to social stability. In this view, the pursuit of (hetero)sexual satisfaction is not evidence of the rise of narcissistic individualism, but rather depended on older notions of character and duty that nevertheless formed the cornerstones of democratic citizenship. In locating initiatives in counselling within the context of the recently founded welfare state, this scholarship emphasises the distinctive elements of British therapeutic culture, demonstrating the importance of national context in determining the specific manifestations of the ‘psychological turn’ apparent across Europe and North America.

Where do magazine advice columnists fit into this picture? Here, I first chart approaches to desire and pleasure in marital, premarital and extramarital sex on the problem page of leading weekly magazine *Woman’s Own* between 1960 and 1990. I then draw on memoirs and oral history interviews with magazine advice columnists to explore how they saw the role, and their changing relationships to psychological expertise. In giving advice on sex and emotions, agony aunts often operated in uncharted waters. Caught in the same maelstrom of social change as other citizens, they had to offer responsible guidance while navigating legal boundaries, editorial restrictions and the need to appeal to readers. Advice columns, alongside the life stories and testimonies of their authors, therefore provide an excellent case study of the transmission of psychological language, concepts and expertise within popular culture. In turn, this case study contributes to debates on the causes and effects of the rise of therapeutic culture in Britain, whether and how this intertwined with “permissiveness”, and especially the extent to which the psychologisation of everyday life reflected or fostered value-free individualism.

## Sex, desire and relationships on the *Woman’s Own* problem page

The magazine problem page brought together the voices and experiences of women (and, more rarely, men) of different ages and life stages. Out of the journalistic necessity of providing a good read, each week the page hosted a mixed constituency of people, including adolescents, housewives and older women, and problems relating to friendship, romance, sex, loneliness, ageing and so on. Letters asked for practical information, guidance on moral quandaries or help with interpersonal relationships, and prompted the advice columnist to adopt in turn the roles of human rolodex, trusted intermediary or final court of appeal. As the place where readers spilled out their most intimate troubles, the problem page acted as a ‘social barometer’ of ‘changing mores’—making such columns an invaluable resource for historians of sex, love, relationships and emotions (Jacobs 2001).

As a market-leading title with a diverse readership, *Woman’s Own* is a particularly good vehicle for such explorations. Established in 1932, by the mid-1960s *Woman’s Own* was the second most successful women’s weekly title (after *Woman*), with circulation of 2.1 million copies per issue. It still held this position in the market in the mid-1980s, despite the decline to 1.1 million sales per week (Winship 1987, 166). Aimed at housewives, it was read by women across all age groups and social classes, with the highest percentage of readers among the wives of ‘skilled workers’, and it also reached a large secondary readership of adolescent girls and men (White 1970, 217; Carter 2016, 85; Ballaster et al. 1991, 133).

Like other titles founded in the interwar years, *Woman’s Own* featured an advice column from its inception (Hackney 2016). Notwithstanding cosmetic changes, the format of the problem page remained almost exactly the same between 1960 and 1990. In 1974, a separate half-page dedicated to men’s problems was introduced, but this was reduced to small section of the main page 3 years later and disappeared entirely in 1980. Likewise, the faces on the page barely changed over the years. From 1945, when the magazine’s first agony aunt Leonora Eyles left, the page appeared under the pseudonym ‘Mary Grant’ (Langhamer 2013, 43). In 1979, the page’s header changed to ‘Mary Grant’s Problem Page. Edited by Angela Willans’.1 By 1981, the column had been rebranded ‘The Angela Willans Problem Page’, and so it stayed until the end of our period.2 The stability in the *Woman’s Own* problem page personnel over three decades makes it easier to track change and continuity in problems and responses over time, without the need to factor in the effects of abrupt shifts in the personality at the helm.

The following section explores discussions of desire, pleasure and the quest for sexual satisfaction in premarital, marital and extramarital relationships on the *Woman’s Own* problem page, using selections from a sample of over 900 letters and responses.3 This correspondence illuminates changing manifestations of “permissiveness” and shifting uses of emotional and psychological language, concepts and explanations, in turn providing insight into the sexualisation of everyday life and the psychologisation of sex and relationships. Across the print media, the relaxation of legal restraints against abortion and homosexuality in the late 1960s made it possible (eventually) for editors to print letters on topics that were once taboo, for advisers to respond more openly and for readers to write more explicitly about what troubled them. These shifts coincided with the rise of the Women’s Liberation Movement, and by the mid-1970s, mass-market magazines had started actively to encourage women’s greater assertiveness in personal and sexual relationships, if in depoliticised terms (Tebbutt 2017, 182, 190–92; Cook 2015, 239–40).

The *Woman’s Own* problem page reflects these sweeping changes in societal attitudes to sex and relationships, and in what it was permissible to print. In the early 1960s, printed letters rarely mentioned premarital sex outside the context of unplanned pregnancy and illegitimacy—and, of course, the frequent recurrence of this problem acted as a warning to readers who might be tempted to err. Only 10 years later, sex was accepted as a relatively normal aspect of relationships prior to marriage.4 As cohabitation increased, divorce rates rose and remarriages became more common (Elliott 1991), letters from the late 1970s onwards asked for help navigating the uncharted waters of ex-spouses and blended families (Grant 1975e).5 In the same years, advisors provided sympathetic reassurance on the harmlessness of cross-dressing, put ‘transvestites’ in touch with the Beaumont Society, provided the addresses of useful organisations for queries about gender reassignment and even provided practical guidance on dealing with six o’clock shadow, such as using ‘the heavier, theatrical kind of cover-up foundation’ (Grant 1977a).6 By the late 1980s, Willans was soothing readers’ fears about the risk of HIV and AIDS, and referring to mechanisms of transmission and means of prevention (Willans 1989a).7 This was a far cry from the short, coded responses to (unprinted) letters about venereal disease tucked away in the corner of page in the 1960s (Grant 1965b).

In multiple ways, the problem page testifies to the major social transformation in viewing sex and sexuality as important, valuable and inevitable aspects of behaviour, identity and relationships. The types of problems printed on the page, the more compassionate and less didactic tone of agony aunts and the more precise and explicit language used by supplicants and advisors alike—all are evidence of the reality of ‘the permissive society’ (Cook 2004, 238–40). Indeed, letter-writers and advice columnists were aware that the speed of change had left some feeling unanchored. By the mid-1970s, Grant (1977e) was gently pointing out to correspondents that it was perfectly fine *not* to have sex outside marriage: ‘there’s nothing wrong or shameful about being a virgin!’.8 Yet, while the problem page speaks to a revolution in sexual attitudes and behaviour, the story it tells about moral versus psychological frameworks of explanation, and the intertwining of “permissiveness” with therapeutic culture, is more complex. This is evident when we look at how letter-writers and advisors approached the quest for sexual satisfaction.

### The pursuit of sexual pleasure

From the 1960s onwards, the problem page promoted the view of sex as an important ‘symbol of love’ in happy, stable relationships (Home 1970). Much else changed over the next few decades, but never the advice columnist’s role in urging correspondents to seek sexual pleasure in marriage. A comparison of exchanges from 1966 and 1987 reveals striking similarities.

In the first, a woman married for 2 years and ‘extremely happy except that I still cannot enjoy the intimate side of marriage’ asked for advice on whether ‘feeling the way I do about the sex act’ would prevent her having children (Anon 1966). Grant (1966b) provided immediate reassurance that ‘enjoyment of sexual intercourse has nothing to do with […] conceiving’, but went on to say that ‘I hope you won’t just resign yourself to this lack of pleasure in sexual relations with your husband’, offering to send leaflets and a list of books if the reader provided a stamped addressed envelope, and suggesting that ‘a visit to your doctor or nearest marriage guidance counsellor would also help you a great deal with your problem’. In the second, Willans (1987a) advised a woman living with a ‘kind, boring’ man and having an affair with ‘a much more exciting’ but unreliable man that a ‘poor sex-life in a basically loving relationship’ was better than the alternative—but ‘you don’t need to settle for a poor sex-life’. If the letter-writer sent an s.a.e., Willans could provide a reading list ‘and you’ll soon find there’s no need to stray from your present relationship to find all the excitement you want’. With the right information and outlook, love and sex could be united to strengthen relationships.

In the context of relationships expected to result in marriage, agony aunts viewed sexual impulses as natural and healthy, even while counselling control in the short-term. In doing so, they openly acknowledged the existence and strength of sexual desire, describing long engagements as ‘sexually, a trying test of willpower’ (Grant 1965c) and ‘very testing’ (Grant 1970c). This perspective on the importance of marital satisfaction also led advisors to encourage individuals and couples to plan their future sexual lives as part of preparation for matrimony. In 1966, Grant (1966c) told an 18-year-old whose fiancé did not show affection to talk to a marriage guidance counsellor because the problem ‘must be tackled before you marry if it’s not to lead to misunderstandings later’. A decade later, she pointed an engaged woman ‘embarrassed and nervous’ about going to a family planning clinic towards a leaflet providing a step-by-step account of what to expect on the first visit (Grant 1976f).

Readers also became more proactive over the years; in 1979, a 22-year-old told Grant that she had recently started using tampons, ‘to accustom myself to sexual intercourse’ before her impending marriage, but because she found insertion difficult and ‘slightly painful’, she now dreaded ‘the thought of making love’ (Anon 1979b). Grant (1979b) reassured the woman that worry and tension could explain the problem, suggested she ‘get to know your body intimately so that you can face exactly what it is you’re anxious about at the prospect of penetration’, and offered to send on the free leaflet ‘Afraid To Make Love’. These exchanges demonstrate acceptance of sex as an important part of married life, but always coupled with an emphasis on taking responsibility for sexual activity within a stable relationship.

### Relocating the moral centre

From the late 1960s another trend is evident: displacement from adherence to external moral standards towards resolution according to the individual’s own judgement. This approach was most often, but not exclusively, taken with unmarried people perceived to have fewer, or less binding, responsibilities to others. The exemplar of this ethical stance is found in Mary Grant’s response to a 17-year-old (Anon 1969a) who wrote to the page because she could not stand ‘the slightest petting’ with her boyfriend, partly because she felt that ‘even slight petting is wrong before marriage’. Grant (1969b) affirmed the legitimacy of the letter-writer’s feelings but emphasised the private nature of such decisions, stating that ‘people’s views on petting have more to do with their personal feelings and their own standards, and those of their “partner”, than with rules about what right and what’s wrong’.9

This stance guided advice based on an avowedly realistic appraisal of the situation, which withheld overt moral judgement and instead set out the options available to the correspondent. A few years later, Grant (1973a) told a woman in her early 20s, who had started to experience ‘pains’ and ‘irritability’ after prolonged heavy petting with her boyfriend, that ‘milder petting or complete abstinence’ were clearly not viable alternatives at this stage. The only options were therefore marriage, sexual intercourse without marriage but with birth control or ending the relationship altogether.10 This advice acknowledged sexual desire, did not impose external standards of morality and left the choice of action to the individual.

Complementary to this position, the advice columnist might locate the basis for future action not in external standards, but in her interpretation of the letter-writer’s *own* unrealised emotional orientation to the problem. In the early 1970s, Grant (1973d) told a 16-year-old annoyed that she was no longer a virgin, but insistent that she did not regret having sex with her boyfriend, that ‘your reaction points to the fact that emotionally, it was a mistake […] you deny that you feel regret when regret is what your letter is all about’. She was advised to ‘face this reaction squarely and use it. It could help you to act less impulsively and think more carefully in future’.11 Almost a decade later, Willans (1981b) applied the same logic in her response to a young woman having an affair with a married man who had children:

Your affair is wrong—not because anyone else says so, but because it’s making you feel guilty and afraid of hurting other people. Therefore it’s wrong by *your own standards* of concern for yourself and others. So the only way out of these destructive feelings is to end your association. Painful, yes, but right for you, for him and his family.

In a similar case, Willans (1982a) advised that ‘the best way to stop feeling guilty is to stop doing what makes you feel guilty’. These responses might appear to reinforce older moral standards, but that is not their internal logic. Rather, the advisor met questions about sex with answers about feelings and located the clue to action within the individual’s own emotional reactions to her situation.

### Rationality, responsibility and maturity

Underlying these responses was a set of quite traditional beliefs: that adults were capable of making rational decisions, even about highly emotional matters; that any relationship entailed responsibilities; and that awareness of these responsibilities must form the basis of rational decision-making. These beliefs, evident in advice columnists’ responses over three decades, proved compatible with “permissive” behaviour, including sex outside marriage, birth control, abortion, divorce, cross-dressing and same-sex relationships. This contradiction is more apparent than real. Until the early 1960s, advisors upheld a rigid, externally imposed standard of morality that was also highly pragmatic: in the absence of reliable contraception, legal abortion or access to divorce, and in a society where most women had little capacity to financially support themselves, it made sense to discourage sex outside the bonds of marriage that might result in illegitimate children (Thane and Evans 2012). As social norms shifted, laws were reformed and women gained some economic independence, individuals made choices about their lives within different parameters. With the same pragmatism, advisors now offered context-specific counsel—but always underpinned by that same belief in rationality and responsibility.

Comparison of responses to married women having affairs in the 1960s and 1980s demonstrates this continuity over time. In the 1960s, Grant (1964a) emphasised the letter-writer’s control over her own actions: ‘you are not powerless. Love isn’t something outside yourself that drags you unwillingly this way or that’. She also reminded supplicants of their responsibilities to others: ‘Take a good look at yourself; you’re living on your emotions and risking all the real and valuable things in your life […] Finish with him, and put your heart immediately into caring for all the people who need your love’ (Grant 1966a). The casual reader might find it difficult to spot any substantial differences between this guidance, and Angela Willans’ advice to a correspondent over two decades later (Willans 1989c):

The stress and depression are entirely your choice. By going for an affair to remedy your marriage problem you’ve landed yourself in a no-through road where you and your lover are cheating on everybody, including each other. Where’s the respect and friendship in that? Guide yourself back to your husband and explain what’s gone wrong between you. I’m sure he’ll show some feeling for you if you show some for him.

As this response shows, agony aunts often had little patience with letter-writers who claimed the inability to control their emotions. Mary Grant’s impatience with one such supplicant was clear when she asked, ‘How was it “inevitable” that he became your lover? What’s so inevitable about hurling yourself out of a safe, loving, happy marriage into all this worry and misery?’ (Grant 1979c).

Advice columnists applied the same rules of rationality and responsibility to many different kinds of problems over these decades. Grant (1967) flatly told a separated woman pregnant by her estranged husband, ‘You chose to marry—that carries continuing responsibilities. You chose to make love, and that has now brought about concern for another life’. She did not judge the sexual adventuring of the 27-year-old who had tried and enjoyed ‘a lot of sex games’ with her ex-boyfriend, but now worried about becoming promiscuous, simply stating that ‘Different people mean different things by promiscuous, but plenty of people have short-term relationships on the way to a lasting one’. However, she quickened at the correspondent’s unwillingness to admit her own agency: ‘plenty of people have a strong, lively interest in sex […] But they don’t all rush into compulsive copulation. They use restraint and responsibility in sex—and so can you […] the short answer is—if you don’t want a promiscuous life, don’t lead one’ (Grant 1977c). Likewise, she acidly queried of the 17-year-old who wanted to know whether to start taking the pill ‘in case I end up having sex without meaning to’, ‘wouldn’t it be better to avoid any situations where you can’t exercise your rational choice about having sex or not?’(Grant 1978a).

Advisors responded more sharply to these queries not because of adherence to traditional moral standards, but because the letter-writers appeared to refuse accountability for their actions. This is evident in the very different tenor of replies to supplicants who demonstrated responsibility. In the early 1960s, women who had ended their extramarital affairs themselves, but were crippled by guilt, were praised for ‘having had the strength […] to put an end to a dangerous and immoral situation’ (Grant 1963c). In the mid-1970s, Grant (1976g) gently suggested to a young teenager, who wanted to know how to access the contraceptive pill without seeing her family doctor, that she might not be ready for sex—but she also passed on details of a Brook Advisory Centre (BAC) where the girl could receive help with ‘emotional worries’ as well as contraceptive advice. Quite sweetly, when a 50-year-old woman caring for her elderly parents who ‘wouldn’t think of leaving them’ wanted to know if it was safe to embark on a sexual relationship, given that her last period had been 2 years earlier, Grant (1973c) told her to ‘go ahead, and I hope you’ll be happy in your friendship’.12 And at the end of the 1980s, Willans (1989a) told a woman castigating herself after she had left her husband and then been abandoned by her lover to ‘be less hard on yourself. Nobody’s immune to being conned when they’re in a vulnerable state and it really shouldn’t sentence you to a lifetime of regret and loneliness’. As in earlier responses, however, this kindly stance depended on the supplicant’s recognition of her own guilt, and the fact that the affair had ended.

Above all else, and consistently over time, advisors valued maturity as a character trait. Grant (1963d) told a wife who had fallen in love with another man that her quest for romance and magic was immature; because she had not ‘really grown up’, her marriage had ‘also failed to mature’. The real meaning of living happily ever after was not ‘excitement and wild passion’, but ‘quiet satisfaction, contentment and pride in the loved one’s qualities and achievements’.13 A person who did not realise that ‘anything which brings suffering to other people is wrong’ was ‘not mature emotionally’ (Grant 1963a). Success in marriage was only possible when couples stopped ‘grasping childishly’ at their differences ‘as excuses for escape’ (Grant 1967). Along similar lines, Grant (1964b) told a young woman who had been unfaithful to her fiancé that she needed to ‘grow up a little and learn to give love as well as to receive it’ and suggested a trial separation in the meantime. In the 1970s, when contraception was freely available, the same character flaws were identified in young people who risked pregnancy by not using it: they were ‘selfish’ (Grant 1975d), ‘immature, irresponsible and short-sighted’ (Grant 1976c). This emphasis on rationality, responsibility and maturity was the precondition for advisors’ adoption of context-specific guidance tailored to individual circumstances—but it is also what prevents any interpretation of that counsel as simply promoting “individualism”.

### A practical psychology?

Advice columnists never encouraged the pursuit of pleasure for its own sake, and they always reminded letter-writers of their responsibilities to others. Indeed, throughout these decades the advice they proffered rarely reflects Rose’s (1989, 239, 253–4) view of post-1960s therapeutic culture as characterised by ‘new techniques of self-introspection, modes of self-presentation and vocabularies of the emotions’ and organised around the measure of ‘personal fulfilment rather than community welfare or moral fidelity’. Certainly, some of these elements emerged more strongly over time, including greater emphasis on the expression of emotion and the use of more sophisticated psychological language, but introspection remained in short supply on the page itself. Correspondents might seek personal fulfilment, but advisors did not encourage them to find it at the expense of existing commitments. Even in the 1980s, advisors continued to offer counsel that was ‘often far more pragmatic, ethically conventional and less individualist or introspective’—advice very much in tune with the ‘practical psychology’ that Thomson (2006, 4) identifies as dominant in the early 20th century.

Indeed, at first glance, much of this counsel does not look “psychological” at all. Grant’s (1977d) statement to the woman whose husband demanded sex twice daily exemplifies a certain trend: ‘It doesn’t need a doctor’s help—just ordinary human understanding’. This pragmatism is especially evident in responses around extramarital affairs. In the early 1960s, tempted women were usually told to use common sense and get over it (Grant 1960a). Self-control could save the situation, especially once they realised that further trespass would be ‘irresponsible’: ‘Do be sensible and make up your mind not to see him again. It will not be impossible for you to forget him if you make up your mind to do so’ (Grant 1961; Grant 1963b). In some respects, this advice did not change much into the 1970s: ‘If you take part in affairs of this kind you can’t expect the rules of the game to change for your sake […] So don’t play’ (Grant 1972a). All women embroiled in unhappy marriages and hopeless affairs needed to do was redirect their efforts and emotions:

nothing will turn up, you know that. So why not turn up something for yourself? You could make life more than bearable, perhaps even enjoyable, for yourself, your children and your husband if you tackled the cause of the rows that are blighting your family life […] try to put life and love into your marriage (Grant 1974c).

As the 1980s bedded in, Willans (1982b) sometimes added the recommendation to visit a marriage guidance counsellor, but the substance of the message did not change: ‘finish with him, and stick to it’.14 At this later date, correspondents might already have explored such avenues before writing to the page, as in the case of a young woman who had tried ‘counselling at a youth centre and advice from friends and relatives’ in her quest to resolve her affair with a married man. Nevertheless, Willans (1981b) stuck to the same line: ‘the only way out of these destructive feelings is to end your association’.

Advice columnists did not only emphasise pragmatic, self-directed action in relation to extramarital affairs; responses to most problems took this tack, and this stance went hand in hand with advisors’ avoidance of explicitly psychological explanations for behaviour. Although from the early 1970s onwards, advice columnists occasionally flirted with depth psychology, suggesting that to really tackle the problem, correspondents needed to understand the reasons for their actions, such explanations were extremely rare. They were also invoked almost exclusively in relation to problems of sexual compulsion: the only way ‘to stop this self-destructive behaviour’ was to understand ‘quite what compels you to do it’ (Grant 1971c).15 These responses implicitly invoked unconscious drives and needs as underlying sexual behaviour, while avoiding this technical vocabulary. But such explanations were infrequent, and only rarely were they followed by recommendations to seek out specialist services such as counselling.

This is not because advisors were detached from wider trends in counselling culture. From the first use of Angela Willans’ real name on the page in 1979, she was introduced as ‘the only advice columnist on the Executive of the National Marriage Guidance Council’ (Anon 1979c). By 1990, her printed biography on the page included her associations with BACs,the Eating Disorders Association and the Samaritans (Anon 1990). It is therefore not surprising that, across this period, Grant and Willans encouraged letter-writers to visit marriage guidance counsellors for help with sex-related problems, including enhancing pleasure in relationships, and recovering from affairs.16 In the mid-1970s, Grant (1976b) also encouraged readers to buy particular books or consult book-lists provided by the Marriage Guidance Bookshop.17

In addition to providing adolescents with information about BACs and their services, Mary Grant (1976d, g) sought to educate older readers about counselling services for young people. In 1975, when one mother whose daughter had sought contraceptive advice worried that ‘these centres are rather antiparent’, Grant (1975c) replied: ‘They’re not antiparent—far from it. They welcome mothers, fathers and friends, and give them confidential help. Brook Advisory Centres recently held a “Mothers’ Week” to make this very point. So ring a Centre and ask for an appointment with a counsellor’. After all, the belief that ‘everyone should have the knowledge, and, if wanted, the means to avoid having unwanted children’ was just ‘a different, and very practical, view-point and really nothing to do with fashion but […] with a genuine concern for individuals and mankind’ (Grant 1972c).

Advice that favoured pragmatic self-directed action over depth psychology should not be immediately dismissed as simply not “psychological”. As we have seen, advisors emphasised rationality, responsibility and maturity in their provision of context-specific guidance to individuals—qualities more associated with Edwardian notions of “character” (Collins 2002; Romani 2002) than with late-20th-century “permissive society”. But they are also the personality attributes at stake in prominent mid-century psychoanalytical theories that diagnosed symptoms such as menstrual pain and infertility as psychological problems stemming from women’s failure to adjust to their appropriate adult roles as wives and mothers (Gameiro and Boivin 2017, 394–97). These psychological approaches filtered into “folk wisdom” and continued to be invoked in different contexts and guises for several decades (Swain 2017, 420–22).

Advisors were not psychologically trained but may have taken some of these ideas on board, especially given their overlap with older models of character and duty. In 1976, for example, a girl wrote to ask Mary Grant’s advice because she could not stand kissing and was ‘afraid at the thought of getting married and having sex’ (Anon 1976). Grant (1976d) explained that the girl surely did not ‘like kissing because it’s the first step along the path you fear so much—love, sex, and commitment’ and advised her to talk the problem over with a ‘Brook Centre or any youth advice service’. This response—the equation of fear of sex with immaturity coupled with direction to a counselling service—blends older and newer approaches to sexual problems.

This combination—the need to work within the resources available to readers and the equation of emotional and psychological maturity with adjustment to (gendered) sexual roles—explains both the practical orientation of guidance, and its emphasis on communication. Advisors perceived an important part of their role as to provide information for those too shy to seek outside advice, too embarrassed to talk their partner about sex or simply unsure where to find the relevant support (Raeburn 1985, 134–141). To this end, they wrote their own leaflets to send out to correspondents, and signposted useful books and associated resources on the page (Ironside 1991, 20, 31, 108).18 Many readers, like the adolescents who looked to agony aunts to fill the gaps that other adults had left in the knowledge of ‘the facts of life’, saw the provision of information as the essential work of the page (Anon 1961).19 By educating letter-writers and readers about sex, advice columnists also equipped them to make informed, rational and responsible decisions, and to take control of their lives.

As well as providing information, advisors consistently and emphatically promoted better communication as the main way to resolve sexual problems in relationships. This basic message remained the same over the decades, although framed in different languages and encountered in different contexts. It might mean making active effort to ‘conquer’ shyness and talk to the doctor or another authority about any help required (Grant 1966d). Advisors reassured correspondents that many people found it difficult to talk about intimate problems, ‘but hundreds of people do, and find relief and a solution to their worries. So please do that—it’s the only way’ (Grant 1968a).

Beyond seeking outside help, couples had to learn to put their adult relationship at the centre of their lives, and to build an exclusive bond of mutual trust and respect (Grant 1965a; Grant 1975a). If trust had been lost through infidelity, couples must realise that it was ‘not you on one side of a barrier and him on the other—you’re together against the same barrier […], and together you can remove it’ (Grant 1972b). To build a relationship ‘even better than it was before the crisis’, couples had to share ‘exactly how you feel’ (Willans 1983). To a wife in a sexless relationship, Grant (1970b) emphasised that the first step was always to ‘open up communication with your husband about sex’. To a husband in a similar situation, she insisted, ‘There just has to have been some kind of emotional build-up to it—a drawing apart, a gradual inability to tell each other how you feel. That’s where the answer is’ (Grant 1977b). In short, there was always ‘far more harm done by not talking’ about sexual feelings and ‘letting mystery take over’ (Grant 1977f).

As premarital sex, cohabitation, divorce and remarriages became more common, those unhappy or anxious about their sex lives received similar advice. The young woman who could only reach orgasm through masturbation and not penetrative sex, and consequently worried that she would ‘never have a happy sex life’ with her fiancé, was told to remember that ‘when there are two people involved, and not just you reacting to you, there has to be a great deal of communication about what takes you towards satisfaction and what doesn’t’ (Grant 1974a). In 1974, a woman (Anon 1974) lamented that she had started to feel used by the ‘tricky sex—dressing up and playing at master and maid, teacher and pupil, etc’ preferred by her older lover. Grant (1974b) suggested that perhaps she felt this way because ‘instead of the games being an enjoyable extra dimension to sex, they’re the sum total of feeling expressed between you’. The couple would only discover their ‘deeper feelings’ for each other ‘by talking about it when you’re not playing games’.

A decade later, responding to a woman who felt guilty over her part in her lover’s divorce, Willans (1984) advised talking to ‘your own loved ones, including your partner, and with a detached counsellor, such as a Samaritan’. This problem, with its mess of old and new relationships, as well as the sympathetic tone of the response, definitely belonged to the 1980s rather than the 1960s. Nevertheless, the advice remained the same: talk it over. Readers’ lives revealed the revolution in sex and relationships since the 1960s, but magazine advisors continued to offer more or less the same solutions to their problems.

## From journalists to counsellors: problem pages in the 1990s

The evidence of the problem page perhaps presents us with a confusing picture of the psychologisation of everyday life, even if we broaden our understanding of what “the psychological” meant in this period. Advice columnists were not opposed to sexual or other counselling services, and sometimes enthusiastically promoted specific organisations. However, they did not *consistently* recommend such services; when they did, this advice rarely followed on from an explicitly psychological explanation of the correspondent’s problem; and, out of a range of possible therapeutic options (multiple variants of counselling, psychotherapy or psychoanalysis), marriage guidance counsellors and BACs were their default options. How can we explain this erratic and restricted engagement with psychological culture?

Advisors’ pragmatism was partly an outcome of the restrictions imposed by format. The problem page combined personalised counsel for correspondents with informative diversion for other readers, operating as a ‘semi-public, semi-private’ realm (Hackney 2016, 108). Advice columnists served up their one-to-one responses for a mass audience, and had to entertain as well as to guide and inform—all within tight word limits (Bingham 2012, 53). This alone made it difficult to elaborate on the potential psychological origins of problems. In published memoirs and oral history interviews, advisors also demonstrated awareness that providing counsel in response to a one-off letter was very different from face-to-face encounters that offered opportunities for further questions and discussion (Ironside 1991, 4, 28; Naik 2018). By definition, anyone who wrote to a magazine problem page either could not find what they needed elsewhere or had rejected other resources available to them (Ironside 1991, 31). Advisors therefore focused on advice that letter-writers at least had the potential resources to enact. Arguably, this remained true even as it became more common for agony aunts to hold qualifications in psychology and counselling, and for these to be flagged on the page, from the mid-1980s.

The limitations of the problem page format leads us back to the most important fact: magazine advisors provided counsel, but they were not counsellors. In oral history interviews, former agony aunts emphasised their status as journalists. Virginia Ironside (2018), *Woman*’s agony aunt between 1978 and 1993, explained that the ‘agony aunt is a journalist, and one must never forget that’. She insisted that readers of the page did not want the ‘waffle’ that counsellors, focusing on the problem rather than the reader, were likely to provide. Suzie Hayman (2018), agony aunt for *Essentials* between 1988 and 1993 and and then *Woman’s Own* from 1993 to 2000, recalled one problem page in the early 2000s that appeared under a celebrity’s name, but ‘the letters were clearly being written by a very good psychotherapist, but in psychotherapy-speak. I could read them and say, “Well, that’s really good advice”, but I don’t think, you know, the, the actual, the readers, found them much fun, they were, they were far too dense’. This anecdote underscores the insistence of Anna Raeburn (2018), *Woman*’s agony aunt between 1974 and 1978, that ‘the primary qualification for writing about people’s difficulties, is the ability to communicate’. Any other qualifications, ‘however esoteric or grand or formal they may be, only obtain in mass media if you have the other qualification, which the ability to use words, the ability to move terms around, the ability to communicate’. The demands of good copy held sway.

This is not quite the whole story, however. Looking forward into the 1990s, it is clear that Angela Willans’ involvement with the Marriage Guidance Council, BACs and similar associations presaged an important trend. Until the last quarter of the 20th century, women’s magazine advisors were almost all professional journalists who received no training to perform the role, and had no prior experience in psychology, counselling or the caring professions.20 From the mid-1980s onwards, more advice columnists, on newspapers, women’s magazines and teen magazines, started to seek out counselling qualifications and/or to become more involved with bodies that offered counselling. In turn, just as on ‘The Angela Willans Problem Page’, some magazines started to flag these markers of expertise to readers. The teen magazine *Mizz*, launched in 1985, introduced advice columnist Tricia Kreitman as ‘a qualified psychologist who specialises in sexual and emotional problems. She’s had training with the Family Planning Association and she taught managers and employers how to get on together’ (Anon 1985). Similarly, in 1988 *Essentials* stressed that agony aunt Suzie Hayman had ‘written three books on women’s health and relationships and has worked for both the FPA [Family Planning Association] and Brook Advisory Centres’ (Anon 1988).

The upswing in advice columnists’ formal qualifications and involvement with counselling organisations in part simply reflects the expansion of psychological services and the increased prestige of the “psy” professions in the second half of the 20th century. As ordinary consumers of psychological culture, Raeburn (1985, 93–5) and Ironside had both sought expert help for emotional difficulties before becoming magazine advisors. Ironside (1991, 3) described herself as ‘evangelical about therapy’ when she first started as *Woman*’s advice columnist. In contrast, the memoirs of journalists who began work on the problem pages in earlier decades reveal their efforts, usually sporadic and undirected, to find out more about psychology so that they could provide informed counsel. Faced with abstruse textbooks on abnormal psychology that did not seem particularly helpful for readers’ more run-of-the-mill problems, advisors usually gave up and instead honed their skills through answering letters (Makins 1974, 49–50, 191–2; Patmore 1993, 86). Irma Kurtz (2018), agony aunt for *Cosmopolitan* between 1972 and 2015, insisted in an interview, 'They, they taught me. The letters taught me'.

With the rise of therapeutic culture, advisors could more easily access training aimed at the problems of “normal” life. That so many chose to do so reflects the pressures that advice columnists faced. Marjorie Proops, most famous for her *Daily Mirror* advice column printed from 1971 to 1996, described the ‘heavy responsibility’ of ‘giving people advice and the chances are they are going to take it’. A ‘particularly dreadful problem’ sometimes caused her sleepless nights (Patmore 1993, 257, 269). Remembering the effects of the work on his mother, Claire Rayner (advice columnist for *Woman’s Own* and *The Sun* in the 1970s), her son Jay seemed almost to relive her emotional commitment, becoming stuck on a particular phrase: 'she did get emotionally involved in some of them. She got very emotionally involved in some of them, […] but she did get emotionally involved in them, and, and, that’s right, she did get emotionally involved in them (Rayner 2019).

Advice columnists perhaps felt this burden even more in an era when newspapers and magazines were committed to providing private responses to all supplicants, employing a small staff to do so. In the heyday of print journalism, advice columnists read and oversaw responses to hundreds of letters per week, bearing ultimate responsibility for advice sent out in their names. This inevitably took a toll on the advisor’s own emotions (Loughran 2020).21 As Raeburn (2018) emphasised, 'sometimes your best isn’t enough, and you know that, and I know that, and only a damned fool pretends it’s anything else […] that costs, it should cost if you’re doing it properly'.

As time went on, advice columnists more often perceived such training as part of their essential equipment for the role. In the 1970s, Deidre Sanders edited *Woman’s Own*’s ‘At Your Service’ consumer advice column. When she transferred these skills to the personal advice column, first on the *Daily Star* and then from 1980 on *The Sun*, she undertook courses with the National Marriage Guidance Council and the British Medical Foundation, and for a time belonged to the British Association for Counselling (Sanders 2018). When Suzie Hayman started at *Essentials*, she decided, ‘“I’m going to do this properly”, and so I went to Relate’ for training. She ended up counselling for Relate for over 7 years. This background was immediately evident in her description of the role of agony aunt in terms of ‘the three stages of counselling, which is that you get the story, you then help the person to understand the story, and then you give them options to do something *themselves* about it’ (Hayman 2019).

In her earlier career, Hayman worked at the FPA, where she came into contact with Ann Lovell, a deputy admin manager at the BAC. After an experience of marriage guidance counselling that she found positive, although the marriage did not last, Lovell had taken a counselling course at Middlesex Polytechnic. Hayman suggested Lovell for a post as one of Virginia Ironside’s behind-the-scenes assistants at *Woman*. Lovell continued in this post until she took up the role of advice columnist on the newly launched title *Bella*, where she stayed until 2004. By the late 1980s, it was standard for advice columnists to work closely with what Lovell (2018) described as ‘the big organisations’ like Relate, BAC and the NSPCC, and these links further reinforced the problem page as a site of everyday psychological guidance. Naik (2018), agony aunt for *Just Seventeen* between 1993 and 2003, similarly described the magazine’s ‘close ties to all the teen organisations’, listing the BAC, BMA, FPA and ‘Marie Stopes’ (now MSI Reproductive Choices).

These relationships with counselling organisations sometimes formed a virtuous circle. Nick Fisher started out on *Just Seventeen* as a freelance writer and became the magazine’s agony uncle after he was asked to reply to some letters from girls with boyfriend problems. He stayed in the role from 1987 to 2004, but initially found the work ‘anxious-making’ because ‘I didn’t feel like I had a qualification. I wasn’t a psychotherapist, I wasn’t a doctor, I, you know, I was a journalist who wrote a lot of features on boyfriends’. This prompted him to ‘do a lot of research and get involved with the Brook Advisory Service, and all sorts of, different charities and agencies that dealt with teenagers, to kind of get as much input and information and kind of right, er, right points of view that I could get’. He ‘actually then started writing books about it, you know, because I’d kind of caught up and done the homework’ (Fisher 2018).

These relationships could run in either direction. Tricia Kreitman’s first degree was in psychology and then, after stints as the head of a local health education unit and running a computer training company with her husband, she took a postgraduate medical diploma in psychosexual therapy. Midway through the course, she was offered the post of advice columnist on *Mizz* when, prior to launch, the editorial team asked the FPA to recommend someone. At the magazine, she ended up working closely with the BAC, and went on to become its Chair (Kreitman 2018). Meanwhile, Naik actually trained as a counsellor in the 1990s because she was ‘thinking about becoming a counsellor’, although she never practised (Naik 2018).

It is clear that, in the last decades of the 20th century, journalists working on problem pages were more likely to pursue associations with counselling and counselling organisations, whether as formal training or in terms of seeking information and advice that they could pass on to correspondents. This applied to advice columnists on magazines and newspapers. However, this expertise did not filter onto the page in the same way in each medium. In the words of Kreitman (2018), magazines used advisors’ qualifications as ‘a selling point’. Hayman (2019) explained that her *Essentials* by-line stressed her links to the FPA because ‘it’s the professionalism, it’s actually saying somebody is coming from a background with some knowledge, some information, you know, some authority, as it were’. Yet at precisely the same moment in time, newspaper problem pages were embracing a more ‘hedonistic’ agenda of ‘sexualised entertainment’ and downplaying their educative role (Bingham 2012, 54, 58). If we agree with Thomson (2006, 250) that heightened individualism, unshackled from social, spiritual and moral constraints, characterises the psychological culture of the closing decades of the century, rather than that of the 1960s “permissive moment”, then the overt turn to expertise of magazine problem pages also bucks this trend.

In part, the difference is down to commercial pressures. In earlier decades, the best-known advice columnists might work on either newspapers or magazines, and move between the two. For example, between 1967 and 1972, Marjorie Proops offered an advice column in *Woman*, additional to that paper’s regular page headed by ‘Evelyn Home’.22 In the 1980s, attracted by the ability of newspapers to reach many more millions of readers, as well as by salaries beyond those magazines could offer, the older generation of agony aunts took up permanent residence on newspapers. In this context, broadcasting the psychological qualifications and therapeutic expertise of advisors became a way for magazines to differentiate their problem pages from those in newspapers. Unlike the sexualised attractions that newspaper problem pages offered readers, this psychological turn fitted with the long-standing mission of women’s magazines to provide help, guidance and support for readers—not simply to inform or entertain.

Conversely, perhaps the best evidence for this interpretation comes from Deidre Sanders’ problem page at *The Sun*. Today, it hosts the only behind-the-scenes answering service in the British press, surviving the last equivalent department on a women’s magazine by nearly 30 years. Sanders (2018) insists on it and referred explicitly to her experience on *Woman’s Own* in explaining why she perceives this service as ‘sacrosanct’. She sees herself as ‘bringing the values of the sixties, seventies and eighties, through to the present day, and I’m the only one who’s been able to sort of fight to achieve that’. Those values extend beyond the support she provides to readers. In 1999, when Ann Lovell’s daughter was dying of cystic fibrosis and she could not cope with responding to letters on *Bella*, Sanders offered the support of her own team of assistants to carry out the work—a quiet act of compassion from a bygone era (Lovell 2018).

## Conclusion

What does this exploration tell us about “permissiveness” and the psychologisation of everyday life? The magazine problem page exhibits complex, perhaps even contradictory, trends. We can read “permissiveness” into the diverse sexual problems reported and printed on the page, and the frank language letter-writers and advice columnists used to discuss these problems. Moreover, from the late 1960s advisors more often referred decisions to the supplicant’s own emotional and ethical intuition, providing a realistic assessment of the options available to the letter-writer and leaving the choice of action up to her. This movement did not jettison morality, but relocated it from an abstract, rigid and externalised standard to the context of the individual’s own life and relationships. In many ways radical, this transformation nevertheless depended on advice columnists’ unshakeable belief in the ability and duty of adults to make rational and responsible decisions.

The shift towards greater openness about sex on the problem page was accompanied by increased expressions of emotional understanding. However, this was not the same as an increase in overtly psychological explanations. Advisors continued to emphasise self-control, responsibility and maturity as key components in decision-making around sex. If they displayed more overt sympathy for supplicants than in previous decades and paid more attention to the emotional aspects of problems, they still offered brisk, practical guidance, emphasised the value of communication in broad terms and referred letter-writers to widely known and/or free counselling services, rather than providing more in-depth psychological explanations or a greater menu of therapeutic options.

Between the 1960s and the 1980s, advice columnists also retained their view of sexual intimacy as the glue holding relationships together, rather than sex as a good in and of itself. The model that Chettiar (2016) and Rusterholz (2021) identify as crucial to postwar (sexual) counselling services for young people and married couples survived on magazine problem pages into the 1980s. Indeed, advisors endorsed behaviour often identified as “permissive” precisely *because* they saw sexual happiness as crucial to healthy, stable relationships. By the end of the 1980s, in the cause of upholding this stance, advice columnists often promoted positions unthinkable three decades earlier: that detailed, explicit language was good, because it aided communication and understanding; that sex before marriage could be a useful “trial run”, provided everyone was honest, open and avoided the risk of pregnancy or disease; and that homosexual and heterosexual relationships on the stable, loving model were equally valid.

Looking at sex and relationships on women’s magazine problem pages therefore sheds new light on “permissiveness” and psychological culture. Between 1960 and 1990, the problem page shows greater openness towards sex and displacement of morality from external standards to the individual, and a continued emphasis on self-control and responsibility, and practical guidance that took at best a superficial veneer. These trends were underpinned by a model of sex as an essential part of loving, stable relationships, and the notion, rarely explicitly articulated but always present, that such relationships were essential to social functioning. In the woman’s world of the magazine, before and beyond the 1980s, the problem page does not show the rise of individualism or the pursuit of pleasure above all else—and in turn, this suggests that looking elsewhere, at the experiences of other “ordinary” people, and other groups still marginalised or neglected in histories of therapeutic culture, has the potential to overturn many assumptions about the causes, contents and consequences of the psychologisation of everyday life.

## Data Availability

Data sharing not applicable as no datasets generated and/or analysed for this study.

## Notes

1. Willans worked on the page from 1964. The real identity of ‘Mary Grant’ prior to the 1960s, and the exact date that Willans took over editorial responsibility for the page rather than just contributing to it as a staff journalist, is unknown. For this reason, I refer to Grant and Willans as separate people, and talk about ‘advisors’ on the page, rather than assuming that Grant and Willans were the same person.

2. It is likely that this awkward transition (Mary Grant—‘edited by’—Angela Willans) was intended to reassure long-term readers of continuity in one of the magazine’s most popular pages at a point when sales across the sector continued to decline, but other agony aunts had shed their pseudonyms and become prominent media figures in their own right.

3. I looked at four issues per year (January, April, July and October), for the period 1960–1990.

4. An illustrative contrast is Grant (1960b,1970a).

5. For further examples, see Willans (1981a, 1986, 1990c).

6. For further examples, see Grant (1975b, 1976h, 1978b,c, 1980) and Willans (1982c, 1985b, 1987b, 1990b).

7. For further examples, see Willans (1989b, 1989c, 1990a,d).

8. For further examples, see Grant (1974d, Grant 1976g).

9. For a similar example, see Grant (1974d).

10. For similar forms of advice based on the options available to the individual, see Grant (1975d, 1976c).

11. For a similar problem, see Grant (1974a).

12. This ‘permissive’ advice did not go unchallenged; a few months later, Grant printed a letter from a reader dismayed at the immorality of this endorsement of extramarital sex: Anon (1973b).

13. For a similar problem, see Grant (1969a).

14. For similar advice, see Willans (1982a).

15. For further examples, see Grant (1973b); Willans (1985a).

16. For further examples, see Grant (1965a, 1966b,c, 1971a, 1975a); Willans (1988).

17. For further examples, see Grant (1976e, 1977b).

18. For examples from the problem page, see Grant (1968b, 1971b, 1975a, 1976a).

19. For further examples, see Anon (1962, 1967, 1969b, 1970, 1973a, 1977, 1984).

20. The one exception was Claire Rayner, who had trained as a nurse.

21. See also Makins (1975, 54, 58); Ironside (1991, 51, 86–88, 151–2).

22. The column, an outcome of the absorption of *Woman’s Mirror* by *Woman*, did not replicate the exact format of the ‘Evelyn Home’ page. It went through iterations including ‘Dear Marje’, ‘Counselling in the Round’ and ‘Male Mail’, but tended to focus on fewer problems in more depth.

## References

[R1] Anon . 1961. “My Knowledge Is so Limited.” Woman’s Own. October 7, 1961.

[R2] Anon . 1962. “I’m Unsure of the Facts of Life.” Woman’s Own. April 7, 1962.

[R3] Anon . 1966. “We Want a Child.” Woman’s Own. July 2, 1966.

[R4] Anon . 1967. “She’s Too Embarrassed.” Woman’s Own. January 7, 1967.

[R5] Anon . 1969a. “I Don’t Like Petting.” Woman’s Own. July 5, 1969.

[R6] Anon . 1969b. “I’m Really Scared.” Woman’s Own. July 5 1969.

[R7] Anon . 1970. “The Facts of Life.” Woman’s Own. April 4, 1970.

[R8] Anon . 1973a. “Can You Explain.” Woman’s Own. July 7, 1973.

[R9] Anon . 1973b. “I’m Shocked.” Woman’s Own. July 14, 1973.

[R10] Anon . 1974. “Sex Games.” Woman’s Own. October 5, 1974.

[R11] Anon . 1976. “I Hate Kissing.” Woman’s Own. April 3, 1976.

[R12] Anon . 1977. “All about Periods.” Woman’s Own. October 1, 1977.

[R13] Anon . 1979a. “I Have to Force Myself to Make Love to My Husband.” Woman’s Own. January 6, 1979.

[R14] Anon . 1979b. “It’s Painful.” Woman’s Own. January 6, 1979.

[R15] Anon . 1979c. “Mary Grant’s Problem Page Edited by Angela Willans.” Woman’s Own. July 7, 1979.

[R16] Anon . 1984. “Will It Harm Me.” Woman’s Own. April 7, 1984.

[R17] Anon . 1985. “Here’s the Person Who Can Help You ….” Mizz. April 12, 1985.

[R18] Anon . 1988. “Dilemma. Your Problems Shared.” Essentials November, 1988.

[R19] Anon . 1990. “Angela Willans Problem Page.” Woman’s Own. January 8, 1990.

[R20] Ballaster R. , Beetham M. , Frazer E. , and Hebron S. . 1991. Women’s Worlds: Ideology, Femininity and the Women’s Magazine. Basingstoke: Macmillan.

[R21] Bingham A . 2012. “Newspaper Problem Pages and British Sexual Culture since 1918.” Media History 18 (1): 51–63.

[R22] Carter F . 2016. “A Taste of Honey: Get-Ahead Femininity in 1960s Britain.” In Women in Magazines: Research, Representation, Production and Consumption, edited by Ritchie Rachel , Hawkins Sue , Phillips Nicola , and Kleinberg S. Jay , 183–97. New York and London: Routledge.

[R23] Chettiar T . 2016. “More than a Contract”: The Emergence of a State-Supported Marriage Welfare Service and the Politics of Emotional Life in Post-1945 Britain.” Journal of British Studies 55 (3): 566–91.

[R24] Collins M . 2002. “The Fall of the English Gentleman: The National Character in Decline, c.1918–1970.” Historical Research 75 (187): 90–111.

[R25] Cook H . 2004. The Long Sexual Revolution: English Women, Sex, and Contraception, 1800-1975. Oxford: Oxford University Press.

[R26] Cook H . 2015. “Nova 1965-1970: Love, Masculinity and Feminism, but Not as We Know It.” In Love and Romance in Britain, 1918-1970, edited by Harris Alana and Jones TimothyWillem . Basingstoke and New York: Palgrave Macmillan.

[R27] Crossley N . 2005. Contesting Psychiatry: Social Movement in Mental Health. London: Routledge.

[R28] Elliott B. J . 1991. “Demographic Trends in Domestic Life, 1945-1987.” In Marriage, Domestic Life and Social Change: Writings for Jacqueline Burgoyne (1944-88), edited by Clark David , 85–108. London and New York: Routledge.

[R29] Fisher N . 2018. “Interview by Tracey Loughran: Audio” September 1, 2018.

[R30] Furedi F . 2004. Therapy Culture: Cultivating Vulnerability in an Uncertain Age. London: Routledge.

[R31] Gameiro S. , and Boivin J. . 2017. “The Psychology of Infertility in Reproductive Medicine and Healthcare, c.1940s-2000s.” In The Palgrave Handbook of Infertility in History: Approaches, Contexts and Perspectives, edited by Davis Gayle and Loughran Tracey . London: Palgrave Macmillan.

[R32] Giddens A . 1991. Modernity and Self-Identity: Self and Society in the Late Modern Age. Cambridge: Polity Press.

[R33] Giddens A . 1992. The Transformation of Intimacy: Sexuality, Love and Eroticism in Modern Societies. Cambridge: Polity Press.

[R34] Grant M . 1960a. “He’s a Married Man.” Woman’s Own. April 2, 1960.

[R35] Grant M . 1960b. “He’s Already Married.” Woman’s Own. July 2, 1960.

[R36] Grant M . 1961. “I’m Fascinated by This Man.” Woman’s Own. October 7, 1961.

[R37] Grant M . 1963a. “She Won’t Admit She Was Wrong.” Woman’s Own. January 5, 1963a.

[R38] Grant M . 1963b. “I’m Having His Baby.” Woman’s Own. January 5, 1963.

[R39] Grant M . 1963c. “This Guilt Is Wrecking My Life.” Woman’s Own. July 6, 1963.

[R40] Grant M . 1963d. “I’ve Fallen Out of Love with My Husband.” Woman’s Own. October 5, 1963.

[R41] Grant M . 1964a. “My Love Has Gone.” Woman’s Own April 4, 1964.

[R42] Grant M . 1964b. “I Can’t Be Faithful.” Woman’s Own. July 4, 1964.

[R43] Grant M . 1965a. “She’s So Cold.” Woman’s Own. July 3, 1965.

[R44] Grant M . 1965b. “Mcj.” Woman’s Own. July 3, 1965.

[R45] Grant M . 1965c. “Must We Wait so Long before Getting Married?” Woman’s Own July 3, 1965.

[R46] Grant M . 1966a. “Our Hopeless Affair.” Woman’s Own. January 1, 1966.

[R47] Grant M . 1966b. “We Want a Child.” Woman’s Own. July 2, 1966.

[R48] Grant M . 1966c. “He Won’t Respond.” Woman’s Own. October 1, 1966.

[R49] Grant M . 1966d. “Now He’s Changed.” Woman’s Own. October 1, 1966.

[R50] Grant M . 1967. “My Confusion.” Woman’s Own. October 7, 1967.

[R51] Grant M . 1968a. “He’s Unloving.” Woman’s Own. April 6, 1968.

[R52] Grant M . 1968b. “It’s Very Personal.” Woman’s Own. July 6, 1968.

[R53] Grant M . 1969a. “I’m So Confused.” Woman’s Own. April 5, 1969.

[R54] Grant M . 1969b. “I Don’t Like Petting.” Woman’s Own. July 5, 1969.

[R55] Grant M . 1970a. “Should I Go with Him.” Woman’s Own. January 3, 1970.

[R56] Grant M . 1970b. “I Couldn’t Resist.” Woman’s Own. April 4, 1970.

[R57] Grant M . 1970c. “Afraid of Pregnancy.” Woman’s Own. April 4, 1970.

[R58] Grant M . 1971a. “Should I Confess.” Woman’s Own. January 2, 1971.

[R59] Grant M . 1971b. “He Can’t Make Love.” Woman’s Own. July 3, 1971.

[R60] Grant M . 1971c. “What’s Wrong with Me.” Woman’s Own. October 2, 1971.

[R61] Grant M . 1972a. “So Upset.” Woman’s Own. January 1, 1972.

[R62] Grant M . 1972b. “Sex Barrier.” Woman’s Own. April 1, 1972.

[R63] Grant M . 1972c. “This Shocked Me.” Woman’s Own. July 1, 1972.

[R64] Grant M . 1973a. “Is This Wrong?” Woman’s Own. April 7, 1973.

[R65] Grant M . 1973b. “I Long to Be a One-Man Girl.” Woman’s Own. April 7, 1973b.

[R66] Grant M . 1973c. “Is It Safe?” Woman’s Own. April 7, 1973c.

[R67] Grant M . 1973d. “Was I Right?” Woman’s Own. October 6, 1973.

[R68] Grant M . 1974a. “Unsatisfactory Sex.” Woman’s Own. July 6, 1974.

[R69] Grant M . 1974b. “Sex Games.” Woman’s Own. October 5, 1974.

[R70] Grant M . 1974c. “Won’t Leave His Wife.” Woman’s Own. October 5, 1974.

[R71] Grant M . 1974d. “I Just Pretend.” Woman’s Own. October 5, 1974.

[R72] Grant M . 1975a. “I Can’t Enjoy Sex.” Woman’s Own. January 4, 1975.

[R73] Grant M . 1975b. “Who Can I Contact?” Woman’s Own. April 5, 1975.

[R74] Grant M . 1975c. “Anti-Parent?” Woman’s Own. April 5, 1975.

[R75] Grant M . 1975d. “A Selfish Lover.” Woman’s Own. July 5, 1975.

[R76] Grant M . 1975e. “Broody for His Baby.” Woman’s Own. October 4, 1975.

[R77] Grant M . 1976a. “Miss W.” Woman’s Own. January 3, 1976.

[R78] Grant M . 1976b. “Much Too Shy.” Woman’s Own. January 3, 1976.

[R79] Grant M . 1976c. “Is My Boyfriend Too Young?” Woman’s Own. April 3, 1976.

[R80] Grant M . 1976d. “I Hate Kissing.” Woman’s Own. April 3, 1976.

[R81] Grant M . 1976e. “He Hasn’t Even Tried.” Woman’s Own. July 3, 1976.

[R82] Grant M . 1976f. “I’m Nervous about This Visit.” Woman’s Own. July 3, 1976.

[R83] Grant M . 1976g. “Too Young for the Pill.” Woman’s Own. October 2, 1976.

[R84] Grant M . 1976h. “I Wish He’d Admit It.” Woman’s Own. October 2, 1976.

[R85] Grant M . 1977a. “Six O’Clock Shadow.” Woman’s Own. January 1, 1977.

[R86] Grant M . 1977b. “Breaking My Heart.” Woman’s Own. January 1, 1977.

[R87] Grant M . 1977c. “I Blame Myself for Leading Him On.” Woman’s Own. January 1, 1977.

[R88] Grant M . 1977d. “He Becomes Bad-Tempered.” Woman’s Own January 1, 1977.

[R89] Grant M . 1977e. “I’m Ashamed of Being a Virgin.” Woman’s Own. April 2, 1977.

[R90] Grant M . 1977f. “She’s as Cold as Ice.” Woman’s Own. July 2, 1977.

[R91] Grant M . 1978a. “Just in Case.” Woman’s Own. January 7, 1978.

[R92] Grant M . 1978b. “Practical Help.” Woman’s Own. January 7, 1978.

[R93] Grant M . 1978c. “Secret Urges.” Woman’s Own. April 1, 1978.

[R94] Grant M . 1979a. “I Have to Force Myself to Make Love to My Husband.” Woman’s Own. January 6, 1979.

[R95] Grant M . 1979b. “It’s Painful.” Woman’s Own. January 6, 1979.

[R96] Grant M . 1979c. “Although I Love Him, I Think I’m Wasting My Time.” Woman’s Own. April 7, 1979.

[R97] Grant M . 1980. “Dressing Up.” Woman’s Own. October 4, 1980.

[R98] Hackney F . 2016. “Getting a Living, Getting a Life: Leonora Eyles, Employment and Agony, 1925-1930.” In Women in Magazines: Research, Representation, Production and Consumption, edited by Ritchie Rachel , Hawkins Sue , Phillips Nicola , and Kleinberg S. Jay , 107–24. New York and London: Routledge.

[R99] Hayman S . 2019. “Interview by Tracey Loughran: Audio” January 7, 2019.

[R100] Home E . 1970. “Coming to Terms.” Woman. July 18, 1970.

[R101] Ironside V . 1991. Problems! Problems! Confessions of an Agony Aunt. London: Robson Books.

[R102] Ironside V . 2018. “Interview by Tracey Loughran: Audio”. 29 August 2018.

[R103] Jacobs B . 2001. “Dearly Departed.” Guardian, 26 November 2001.” https://www.theguardian.com/media/2001/nov/26/mondaymediasection2.

[R104] Kreitman T . 2018. “Interview by Tracey Loughran: Audio”. November 29, 2018.

[R105] Kurtz I . 2018. “Interview by Tracey Loughran: Audio”. September 4, 2018.

[R106] Langhamer C . 2013. “Everyday Advice on Everyday Love. Romantic Expertise in Mid-Twentieth Century Britain.” L’Homme 24 (1): 35–52. 10.7767/lhomme.2013.24.1.35.

[R107] Lasch C . 1979. The Culture of Narcissism: American Life in an Age of Diminishing Expectations. New York: W.W. Norton & Co.

[R108] Lewis J. , Clarke D. , and Morgan D. . 1991. Whom God Has Joined Together: The Work of Marriage Guidance. London: Routledge.

[R109] Loughran T . 2020. “The Most Helpful Friends in the World’: Letters Pages, Expertise and Emotion in British Women’s Magazines, c.1960-1980.” In Women’s Periodicals and Print Culture in Britain, 1940s-2000s: The Postwar and Contemporary Period, edited by Forster Laurel and Hollows Joanne , 133–49. Edinburgh: University of Edinburgh.

[R110] Lovell A . 2018. “Interview by Tracey Loughran, Audio” September 24, 2018.

[R112] Makins P . 1975. The Evelyn Home Story. Glasgow: Fontana/Collins.

[R111] McKay S . 2008. “Advice Columns as Cultural Intermediaries.” Australian Journal of Communication 35 (no.3): 93–103.

[R113] Mort F . 2011. “The Permissive Society Revisited.” Twentieth Century British History 22 (no.2): 269–98.

[R114] Naik A . 2018. “Interview by Tracey Loughran, Audio”. September 14, 2018.

[R115] Osborne T . 1993. “Mobilizing Psychoanalysis: Michael Balint and the General Practitioners.” Social Studies of Science 23 (1): 175–200. 10.1177/030631293023001006.

[R116] Patmore A . 1993. Marje: The Guilt and the Gingerbread. London: Sphere Books.

[R117] Raeburn A . 1985. Talking to Myself. London: Sphere Books.

[R118] Raeburn A . 2018. “Interview by Tracey Loughran, Audio”. August 31, 2018.

[R119] Rayner J . 2019. “Interview by Tracey Loughran, Audio”. January 21, 2019.

[R120] Rieff P . 1966. The Triumph of the Therapeutic: Uses of Faith after Freud. New York: Harper and Row.

[R121] Romani R . 2002. National Character and Public Spirit in Britain and France, 1750-1914. Cambridge: Cambridge University Press.

[R122] Rose N . 1989. Governing the Soul: The Shaping of the Private Self. London and New York: Routledge.

[R123] Rusterholz C . 2019. “‘You Can’t Dismiss That as Being Less Happy, You See It Is Different’. Sexual Counselling in 1950s England.” 20 Century British History 30 (3): 375–98. 10.1093/tcbh/hwz008. 30986822

[R124] Rusterholz C . 2021. “‘If We Can Show That We Are Helping Adolescents to Understand Themselves, Their Feelings and Their Needs, Then We Are Doing [a] Valuable Job’: Counselling Young People on Sexual Health in the Brook Advisory Centre (1965-1985).” Medical Humanities. 10.1136/medhum-2021-012206. 23 Aug 2021. PMC10359536 34426537

[R125] Rusterholz C . 2022. “Youth Sexuality, Responsibility, and the Opening of the Brook Advisory Centres in London and Birmingham in the 1960s.” Journal of British Studies 61 (2): 315–42. 10.1017/jbr.2021.188.

[R126] Sanders D . 2018. “Interview by Tracey Loughran, Audio”. September 18, 2018.

[R127] Swain S . 2017. “The Interplay between Infertility and Adoption Practice in Twentieth-Century Australia.” In The Palgrave Handbook of Infertility in History: Approaches, Contexts and Perspectives, edited by Davis Gayle and Loughran Tracey . London: Palgrave Macmillan.

[R128] Tebbutt M . 2017. “From ‘Marriage Bureau’ to ‘Points of View’: Changing Patterns of Advice in Teenage Magazines: Mirabelle, 1956-77.” In Peoples, Places and Identities: Themes in British Social and Cultural History, 1700s-1980s, edited by Kidd Alan and Tebbutt Melanie . Manchester: Manchester University Press.

[R129] Thane P. , and Evans T. . 2012. Sinners? Scroungers? Saints? Unmarried Motherhood in Twentieth-Century England. Oxford: Oxford University Press.

[R130] Thomson M . 2006. Psychological Subjects: Identity, Culture and Health in Twentieth-Century Britain. Oxford: Oxford University Press.

[R131] Weeks J . 2017. Sex, Politics and Society: The Regulation of Sexuality since 1800. 4th edn. London: Routledge.

[R132] White C . 1970. Women’s Magazines, 1693-1968. London: Michael Joseph.

[R133] Willans A . 1981a. “This Means We Can’t Have Children.” Woman’s Own. January 3, 1981.

[R134] Willans A . 1981b. “We’re Both Worried.” Woman’s Own. April 4, 1981.

[R135] Willans A . 1982a. “What Do I Want.” Woman’s Own April 3. 1982.

[R136] Willans A . 1982b. “I Don’t Know Why I Do It.” Woman’s Own. July 3, 1982.

[R137] Willans A . 1982c. “I Still Feel Guilty.” Woman’s Own. October 2, 1982.

[R138] Willans A . 1983. “Has He given up His Other Woman.” Woman’s Own. April 2, 1983.

[R139] Willans A . 1984. “Plagued by Guilt.” Woman’s Own. October 6, 1984.

[R140] Willans A . 1985a. “Jealous of His Wife.” Woman’s Own. July 6, 1985.

[R141] Willans A . 1985b. “He Has to Dress Up.” Woman’s Own. October 5, 1985.

[R142] Willans A . 1986. “What Do I Tell the Kids.” Woman’s Own. July 5, 1986.

[R143] Willans A . 1987a. “I Want to Change My Sex-Life.” Woman’s Own. January 3, 1987.

[R144] Willans A . 1987b. “He Wants Me to Beat Him.” Woman’s Own. October 3, 1987.

[R145] Willans A . 1988. “How Can I Forget Him.” Woman’s Own. January 2, 1988.

[R146] Willans A . 1989a. “I’ll Never Trust Anyone Again.” Woman’s Own. April 4, 1989.

[R147] Willans A . 1989b. “I’m Scared I’ve Got VD.” Woman’s Own. July 3, 1989.

[R148] Willans A . 1989c. “He Doesn’t Care.” Woman’s Own. October 2, 1989.

[R149] Willans A . 1990a. “Could It Be AIDS.” Woman’s Own. July 2, 1990.

[R150] Willans A . 1990b. “Is This Wrong.” Woman’s Own. July 2, 1990.

[R151] Willans A . 1990c. “Should I Keep Them Apart?” Woman’s Own October 1, 1990.

[R152] Willans A . 1990d. “I Can’t Trust Her.” Woman’s Own. October 1, 1990.

[R153] Winship J . 1987. Inside Women’s Magazines. London: Pandora.

[R154] Wright K . 2008. “Theorizing Therapeutic Culture: Past Influences, Future Directions.” Journal of Sociology 44 (no.4): 321–36.

